# Large animal models in experimental knee sports surgery: focus on clinical translation

**DOI:** 10.1186/s40634-015-0025-1

**Published:** 2015-04-15

**Authors:** Henning Madry, Mitsuo Ochi, Magali Cucchiarini, Dietrich Pape, Romain Seil

**Affiliations:** Center of Experimental Orthopaedics, Saarland University Medical Center and Saarland University, Bldg 37, Kirrbergerstr. 1, D-66421 Homburg, Germany; Cartilage Net of the Greater Region, Homburg, Germany; Department of Orthopaedic Surgery, Saarland University Medical Center and Saarland University, D-66421 Homburg/Saar, Germany; Department of Orthopaedic Surgery, Graduate School of Biomedical Sciences, Hiroshima University, Hiroshima, Japan; Department of Orthopaedic Surgery, Centre Hospitalier du Luxembourg, L-1460 Luxembourg, Luxembourg; Sports Medicine Research Laboratory, Public Research Centre for Health, Luxembourg, Centre Médical de la Fondation Norbert Metz, 76 rue d’Eich, L-1460 Luxembourg, Luxembourg

**Keywords:** Musculoskeletal disorders, Large animal models, Sheep, Paediatric anterior cruciate ligament reconstruction, High tibial osteotomy, Articular cartilage repair, Osteoarthritis, Clinical studies, Mesenchymal stem cells, Magnetic-assisted delivery

## Abstract

Large animal models play a crucial role in sports surgery of the knee, as they are critical for the exploration of new experimental strategies and the clinical translation of novel techniques. The purpose of this contribution is to provide critical aspects of relevant animal models in this field, with a focus on paediatric anterior cruciate ligament (ACL) reconstruction, high tibial osteotomy, and articular cartilage repair. Although there is no single large animal model strictly replicating the human knee joint, the sheep stifle joint shares strong similarities. Studies in large animal models of paediatric ACL reconstruction identified specific risk factors associated with the different surgical techniques. The sheep model of high tibial osteotomy is a powerful new tool to advance the understanding of the effect of axial alignment on the lower extremity on specific issues of the knee joint. Large animal models of both focal chondral and osteochondral defects and of osteoarthritis have brought new findings about the mechanisms of cartilage repair and treatment options. The clinical application of a magnetic device for targeted cell delivery serves as a suitable example of how data from such animal models are directly translated into in clinical cartilage repair. As novel insights from studies in these translational models will advance the basic science, close cooperation in this important field of clinical translation will improve current reconstructive surgical options and open novel avenues for regenerative therapies of musculoskeletal disorders.

## Introduction

Large animal models play a crucial role in orthopaedic sports surgery, as they are critical for the exploration of new experimental strategies and the clinical translation of novel techniques (Espregueira-Mendes & Karahan 2014). On December 3^rd^, 2014, about 100 clinicians and basic scientists from Europe, North America, and Asia, met on the occasion of the Research Day of the Annual Congress of the *Société Française d’Arthroscopie* in Luxembourg to discuss requirements for such translational animal models (Table 1). This research day was organized together with the Cartilage Net of the Greater Region, a multinational forum for exploring basic scientific and translational research, development and clinical applications in the diverse field of articular cartilage.

**Table 1:** **Lectures of the Research Day session on December 3** ^**rd**^ **, 2014 of the Annual Congress of the** ***Société Française d’Arthroscopie*** **in Luxembourg**

**Speaker**	**City**	**Country**	**Title of lectures**
Magali Cucchiarini	Homburg	Germany	Recombinant adeno-associated viral vectors as efficient tools for musculoskeletal gene therapy
Christel Henrionnet	Vandoeuvre lès Nancy	France	Innovative and non-invasive evaluation of the quality of collagen scaffold functionalized by human mesenchymal stem cells before graft in cartilage lesion
Henning Madry	Homburg	Germany	Animal models for cartilage repair
Didier Mainard	Vandoeuvre lès Nancy	France	Hoffa ligament: a fat tissue with pro-inflammatory properties
Mitsuo Ochi	Hiroshima	Japan	Cartilage repair using magnets
Dietrich Pape	Luxembourg	Luxembourg	The preclinical sheep model of high tibial osteotomy
Astrid Pinzano	Vandoeuvre lès Nancy	France	Bioengineering and cartilage
Romain Seil	Luxembourg	Luxembourg	Large animal models for pediatric ACL reconstruction

The purpose of this contribution is to provide critical aspects of relevant animal models in experimental knee sports surgery, including paediatric anterior cruciate ligament (ACL) reconstruction, high tibial osteotomy, and articular cartilage repair. These three models discussed are based on the individual presentations (Table 1) and thought to provide a representative and clinically relevant, although by no means exhaustive selection of translational animal models in this field. This paper is aimed at the diverse community of those engaging in experimental orthopaedics, among which orthopaedic surgeons, biomaterial scientists, cell and molecular biologists, biochemists, biotechnologists, and biomechanical engineers. It intends to highlight new developments and to display how far knowledge gathered from these models is already (or will be in the near future) translatable in humans. An emphasis is placed on the “transferability” to patients.

### General requirements for translational animal models in experimental orthopaedics and sports medicine

General requirements for large animal experiments in translational orthopaedic research are manifold: 1) the model needs to be comparable to the human species, 2) comparison data need to be available, 3) the results must be transferable, 4) surgery must be technically feasible, 5) a specialized animal facility must be available with acceptable related expenditures, 6) animals need to be affordable and available, 7) surgery should be ethically accepted, and finally 8) anaesthesia, surgery, and rehabilitation need to be tolerated by the selected animals. Nevertheless, for the human knee joint, no gold standard exists, as the models differ in many important structural and functional aspects compared with the clinical situation (Poole et al. 2010). There is no large animal model replicating the human knee joint perfectly (Aigner et al. 2010). In addition, the activity pattern of animals is different compared with the bipedal locomotion of humans. For example, their quadruped gait results in altered biomechanics compared to the human knee (Rudert et al. 2000), together with differences in the knee range of motion and resting positions. Also, the biomechanical properties of knee joint cartilage are species-specific (Simon 1971), resulting in significant differences of the mechanical properties (Athanasiou et al. 1991).

## Review

### Large animal models for paediatric anterior cruciate ligament reconstruction

The treatment of ligamentous ACL lesions in the paediatric population causes a therapeutic dilemma for physicians because of the lack of current international guidelines. To overcome this problem, a variety of experimental studies concerning the growth plates and paediatric ACL reconstruction have been performed in small and large animal models (Edwards et al. 2001; Guzzanti et al. 1994; Janarv et al. 1998; Ono et al. 1998; Meller et al. 2009a; Meller et al. 2009b; Meller et al. 2008a; Meller et al. 2008b; Seil et al. 2008; Stadelmaier et al. 1995). The complexity of paediatric ACL reconstruction makes the interpretation of this surgical technique and the transferability of its results to humans difficult when small animal models are used. However, there are strong similarities between the stifle joint and the human knee joint. From an anatomical point of view, these joints have asymmetric collateral as well as cruciate ligaments and menisci and the articulating surface is covered with articular cartilage (Dye 1987; Arnoczky & Marshall 1977; Bosch & Kasperczyk 1993). Based on our own experiments (Seil et al. 2008), the sheep model seems to be appealing for the above-mentioned study purpose. Merino sheep are easily available, they are easy to handle and are ethically better accepted than dogs or primates (Allen et al. 1998). Three gross anatomic features have the potential to interfere with ACL surgery and must be known by the surgeon to avoid potential pitfalls: the very narrow intercondylar notch of this species, the different -much more undulating- form of the distal femoral growth plate in comparison to the human knee and the range of motion of the stifle joint with spontaneous 30-40° extension deficit.

Paediatric ligamentous ACL injuries do rarely occur before the age of 8 or 9 years (Chotel et al. 2013). Therefore, a critical point to perform ACL reconstructions in growing sheep is the fact that surgery should be performed at a moment corresponding to the age where ligamentous ACL injuries in children appear to be the most frequent and problematic. In their early growth studies in humans, Bailey and Pinneau showed that at a skeletal age between 8 and 12 years, humans reach 79-92% of their adult height in females and 72-83% in males (Bayley & Pinneau 1952). In the absence of precise data from veterinary studies, sheep should therefore ideally be operated when they reach between 70% and 90% of the adult height. However, information on animals is rarely available (Salomon 2001). Studies on the longitudinal growth of the sheep forearm revealed that they reached 70% of their adult height at day 20 and 90% at day 167 (Salomon 2001). Supposing that these data could be transferred to the hind limb of this species, the use of 4-months old sheep appears justified, and grossly 4 cm of longitudinal hind limb growth could be expected at this age (Seil 2003). This sheep model was used to evaluate the risk for growth disturbances of transphyseal drilling and ACL reconstruction (Seil et al. 2008). Three technical variants were used. In group I, the ACL was resected and 5-mm tunnels were drilled and left empty in four-months-old Merino sheep. Unilateral ACL reconstruction using an autologous Achilles tendon graft and rigid button fixation was performed in group II. A double-stranded graft with a diameter of 5 mm was used in group II-A, and a single stranded graft with a diameter of 3 mm in group II-B. The tunnel diameter was 5 mm in both groups. Six months after the procedure, the combination of peripheral, posterolateral growth plate injuries and empty tunnels led to severe growth deformities on the femoral side with a shortening of the lateral femur of 8 mm (7–10 mm), a valgus deformity of 12.8° (12-14°) and a flexion deformity of 8.6° (5-15°). Histological examination revealed a strong bone bridge formation over the physis and an injury to the perichondral structures. Central growth plate lesions on the tibia did not induce growth abnormalities. Transphyseal ACL replacements did not cause clinically relevant growth disturbances, neither on the tibia, nor on the femur, even if the drilling injury damaged the perichondral structures on the posterolateral physis.

### Clinical relevance for paediatric anterior cruciate ligament reconstruction

This study showed that the sheep model is useful to analyze the outcome of paediatric ACL reconstruction with open growth plates. In terms of risk for growth changes, we identified major differences between femoral and tibial growth plate injuries caused by tunnel drilling. On the tibial side the central positioning of the drill hole in the proximal tibial growth plate appeared to be safe in terms of growth abnormalities. On the femoral side, however, the eccentrically placed femoral tunnel is at risk to injure the periphery of the growth plate. In the paediatric knee, this may either occur during the drilling procedure, for instance if the posterior wall of the tunnel gets injured (posterior blow-out) or if the surgeon chooses to perform an extraepiphyseal graft placement during which this zone might be rasped for better graft adherence to the bone (Kocher et al. 2002). Injuries of the perichondral structures have shown to be at risk to develop major growth abnormalities due to an asymmetric remaining growth (Koman & Sanders 1999; Robert & Casin 2010; Kocher et al. 2002; Chotel & Seil 2013). In the Ogden classification, they are classified as type VI injuries and correspond to a localized peripheral bone bridge (Ogden 1981).

Furthermore, large animal models showed that filling the tunnels with tendon grafts prevented growth abnormalities in transphyseal ACL replacement procedures. Several other studies confirmed the suitability of this model for the study of paediatric ACL reconstruction (Meller et al. 2009a; Meller et al. 2009b; Meller et al. 2008a; Meller et al. 2008b).

### The preclinical sheep model of high tibial osteotomy

HTO is an excellent alternative to knee arthroplasty, especially for younger and physically active patients with knee OA of the medial tibiofemoral compartment and varus malalignment (Brinkman et al. 2008; Pape et al. 2010; Lobenhoffer & Agneskirchner 2003a; Lobenhoffer & Agneskirchner 2014; W-Dahl et al. 2012; Prodromos et al. 2015). The weightbearing axis is shifted away from the medial compartment. Consequently, the load distribution between the medial and lateral compartments of the knee is altered (Van Thiel et al. 2011). Loading is transferred towards the lateral tibiofemoral compartment -especially when a valgus overcorrection is performed- and loading of the medial compartment is decreased (Agneskirchner et al. 2007). Preclinical large animal models of HTO can be used to test different types of osteotomies and to evaluate the effects of lower limb alignment on the reconstructive therapy of articular cartilage lesions and on the development and progression of OA. Traditionally, our experimental knowledge about osteotomies was chiefly based on cadaver studies (Agneskirchner et al. 2007; Lim et al. 2011), models of below-knee amputation or femur valgus osteotomy in guinea-pigs (Wei et al. 2001), on data from cartilage samples obtained from patients undergoing total knee replacement (Otsuki et al. 2008; Wei et al. 2001) and on CT-osteoabsorptiometric investigations in patients (Muller-Gerbl et al. 1992; Madry et al. 2010). More recently, the sheep has emerged as a suitable preclinical animal model of HTO (Pape & Madry 2013). In these studies, adult Merino sheep underwent an HTO of their tibiae. Either a medial open-wedge technique inducing a normal and an excessive valgus alignment or a closed-wedge technique inducing a varus alignment were applied (Table 2). Relevant steps of the surgical technique were essentially performed according to the clinical recommendations of osteotomy experts (Lobenhoffer & Agneskirchner 2003b). During the course of the surgeries, no intraoperative complications occurred, and the popliteal artery and femoral vein were never injured. Some species-specific complications emerged in the post-operative course (Table 3). These included instability of the osteotomy which occurred as a consequence of performing a classical monocortical fixation of the three proximal screws of the implant as in patients. Also, chronic patella dislocation was seen, caused by excessive valgus osteotomy and subsequently insufficient closure after medial arthrotomy, and intraarticular infection. Taken these specific complications into account, the following surgical principles were identified as a prerequisite for solid bone healing and the maintenance of the correction in sheep: 1) a medial and longitudinal approach to the proximal tibia, 2) biplanar osteotomy to increase the initial rotatory stability regardless of the direction of correction (Pape et al. 2011), 3) application of a small, narrow but long implant with locking screws (e.g. small stature HTO plate; Synthes, Umkirch, Germany), 4) posterior plate placement to avoid slope changes, and 5) use of bicortical screws to account for the brittle bone of the tibial head and to avoid tibial head displacement (Tables 2 and 3). Thus, although successful HTO in sheep is complex and requires attention to these principles, the sheep represents because of its similarities with humans (Allen et al. 1998) an elegant model to induce axial malalignment in a clinically relevant environment, and osteotomy healing under challenging mechanical conditions (Pape & Madry 2013).

**Table 2:** **Comparison of surgical anatomical parameters for HTO in sheep and humans (adapted from** (Pape & Madry 2013) **with permission)**

**Congenital anatomical differences**	**Sheep stifle joint**	**Human knee**	**Surgical consequence for the sheep HTO model**
Tibial plateau width [mm]	46-56	60-70	Match screw length
			Narrow and strait plate design necessary
Tibia valga [°]	3.5	0	Valgus overcorrection more likely
Normal knee range of motion (transverse axis) [°]	0-35-72	0-0-140	Dorsal plate positioning after open wedge HTO is recommended due to increased loading of the posterior tibial plateau
tibial tuberosity dimension adding to the AP diameter of the tibial head [%]	30-35	10-15	Anterior plate misplacement more likely
Tibial tuberosity height distance in relation to joint line [mm]	10-15 mm	25-30 mm	Anterior plate misplacement more likely
Posterior slope of the posterior articular surface [°]	20 ± 3	0-10	Narrow and straight plate design necessary for posterior placement
Biomechanical properties of tibial head	Brittle cortical bone, together with little spongious bone	Elastic cortical bone with an exuberant amount of spongious bone	Bicortical proximal screw placement mandatory to avoid fracture/dislocation
			Biplanar osteotomy mandatory regardless of the desired direction of correction
Musculature of the hind limb	Voluminous on medial and lateral side of the femur	Remote from bony knee structures	Distal femoral and proximal lateral tibial osteotomy almost impossible to conduct, stay on the medial side of the proximal tibia for any desired correction angle
Trochlea ridge	Medial ridge extending further cranially and dorsally than lateral ridge	Lateral ridge extending further laterally and anteriorly	Higher propensity of patella instability after valgus correction

**Table 3:** **Comparison of probabilities of potential pitfalls among sheep and humans (adapted from** (Pape & Madry 2013) **with permission)**

**Structure involved**	**Human**	**Sheep**
Neurologic injuries	++	NR
Osteonecrosis of proximal fragment	++	NR
Fracture through proximal fragment with violation of joint space	++	NR
Infection	+	+++
Vessel injury	+	++
Subchondral bone cysts (tibial head) without screw perforation	NR	+
Compartment syndrome	+	NR
Non-union	+	NR
Loss of correction due to implant failure	#	+
Overcorrection	#	NR

“NR” not reported; + seldom, ++ infrequent, +++ frequent, # may depend on implant design (Song et al. 2010).

### Clinical relevance for high tibial osteotomy

Published studies using this model have focused so far on the effect of axial alignment on the lateral tibiofemoral compartment. Specifically, effects on the articular cartilage and the development of OA, the lateral meniscus and the subchondral bone were investigated (Madry et al. 2013b; Madry et al. 2014b; Ziegler et al. 2013; Ziegler et al. 2014). The data first revealed interesting and specific topographical relationships that are present in the central region of lateral tibiofemoral compartment, regardless of correction. For example, there exists a correlation between the thickness of the articular cartilage layer and the subchondral bone. The important protective role of the lateral meniscus for the cartilage (Beaufils et al. 2006) is reflected in the correlation of thickness between the articular cartilage in the submeniscal periphery and the lateral meniscus. The clinical observation that cartilage lesions proceed much faster after lateral than after medial meniscectomy, and that the clinical outcomes of lateral meniscectomy are significantly worse than after medial meniscectomy underscore the delicate balance between the lateral meniscus and the articular cartilage (Bolano & Grana 1993; Hoser et al. 2001; Macnicol & Thomas 2000; Scheller et al. 2001; Heijink et al. 2012).

As valgus HTO leads to an increase in the pressure in the lateral compartment, the subsequent question was whether HTO results in structural changes in the lateral tibiofemoral osteochondral unit and lateral meniscus, and whether these changes depend in the extent of correction; reflective of the pressure in the lateral compartment. Importantly, the pressure increase in the lateral compartment following standard correction valgus HTO (with 4.5° tibial valgus) did not lead to mid-term morphological alterations in the lateral tibiofemoral compartment. In contrast, the higher increase in pressure following valgus overcorrection (with 9.5° tibial valgus) induced adaptive subchondral bone changes, reflected by an increased specific bone surface (BS/BV) in the subarticular spongiosa compared with unloading by varisation. Also, in the lateral menisci, a decrease in the number of cells in the red–red (peripheral) zone of the middle third was noted, however without structural changes (such as meniscal lesions). The lateral meniscus (Beaufils et al. 2006) is of specific importance, since the peak contact stress and maximum shear stress in the cartilage increased 200% more after a lateral than a medial meniscectomy under axial femoral compressive loads (Pena et al. 2006). Altogether, these results show that for the clinical situation, opening wedge HTO is a safe surgical procedure for the lateral tibial osteochondral unit and the lateral meniscus.

### Animal models for cartilage repair

**Figure 1:**
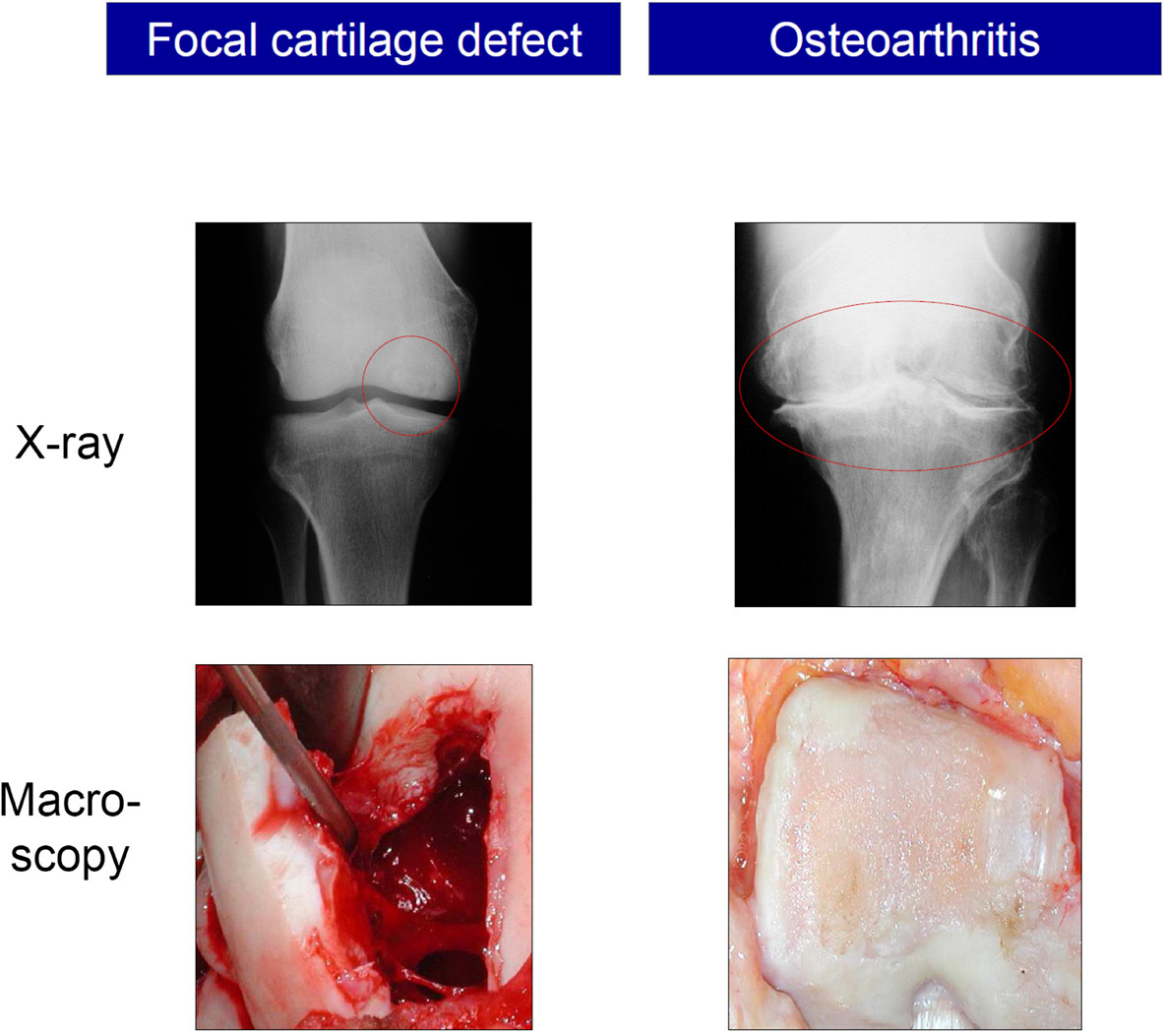
**When contemplating on the use of animal models of articular cartilage defects, the important difference between focal, non-OA cartilage defects and OA lesions needs to be kept in mind.** Focal defects are usually surrounded by a normal cartilaginous tissue (left side, shown is a lesion caused by osteochondritis dissecans of the human knee). The often ill-defined OA lesions are often larger in size and may affect the entire joint (right side, shown is a case of tricompartimental osteoarthritis of the human knee).

Animal models for articular cartilage repair are vital for basic scientific and translational studies (Hunziker 2009; Blaney Davidson et al. 2014). They need to reflect the different appearances and aetiologies of cartilage defects, which may be caused by OA, trauma, osteochondritis dissecans, and osteonecrosis (Madry et al. 2010). Such models may be used to study spontaneous cartilage repair or improved reconstructive surgical options. These currently include the removal or refixation of chondral or osteochondral fragments, autologous or allogeneic osteochondral transplantation, marrow stimulation, articular chondrocyte implantation, or HTO (Hunziker 2002; Madry et al. 2011). Importantly, the difference between focal, non-OA cartilage defects and the often ill-defined OA lesions needs to be appreciated (Figure 1). Focal defects are usually surrounded by a normal cartilaginous tissue, while OA lesions are often larger in size and thus may affect the entire joint surface and are of different depths (Madry et al. 2011; Pritzker et al. 2006). Animal models also play an important role in evaluating novel experimental treatments (Cucchiarini et al. 2014), such as application of bioinspired scaffolds or defined cell populations (Henderson & La Valette 2005; Deie et al. 2007; Bosch & Kasperczyk 1993; Frisbie et al. 2006b).

**Figure 2:**
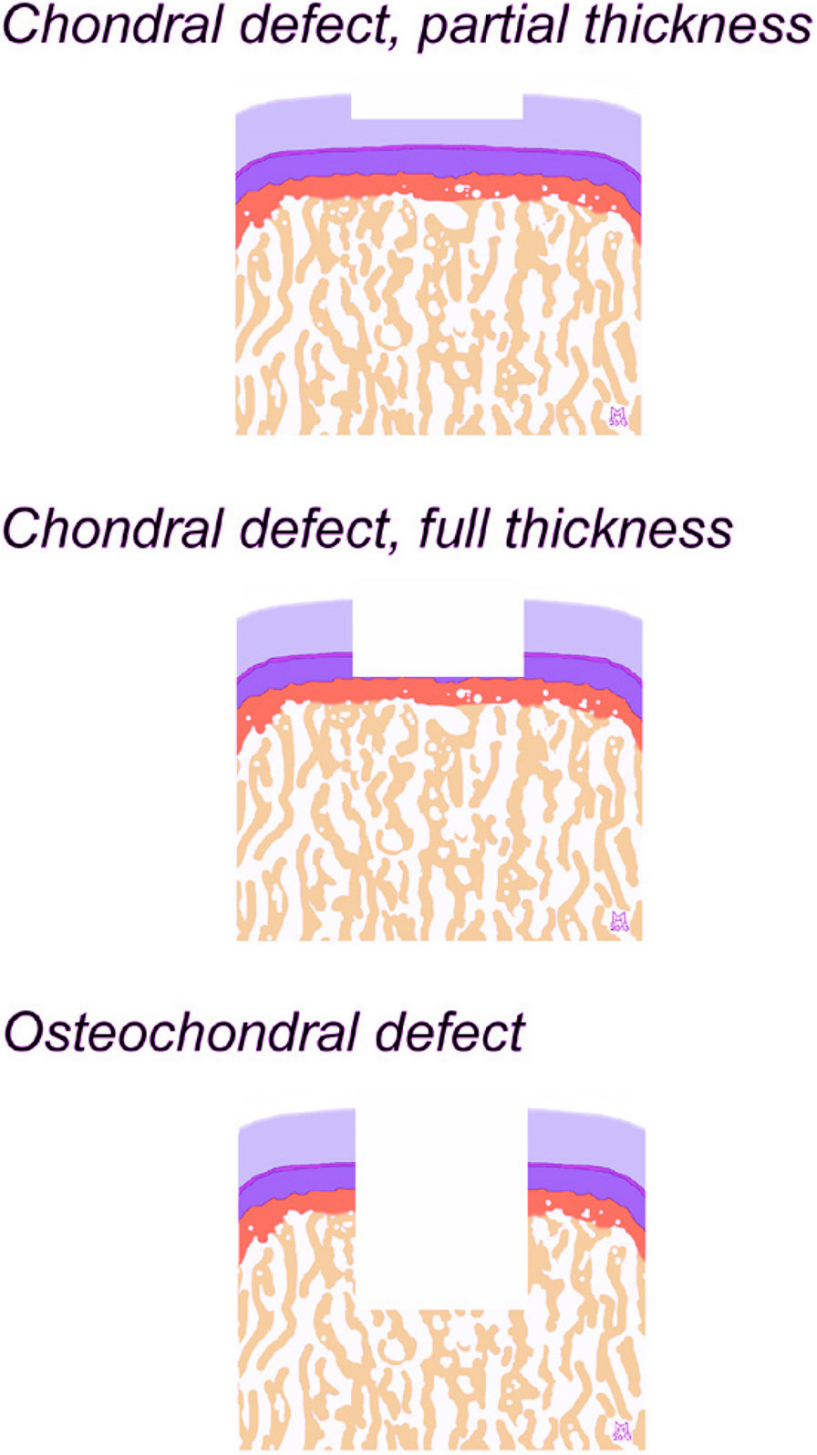
**Classification of cartilage defects.** Both partial- and full-thickness chondral defects involve, by definition, only the cartilage layer. Osteochondral defects extend through the subchondral bone plate into the subchondral bone. Light blue: articular cartilage; dark blue: calcified cartilage; dark orange: subchondral bone plate; orange: subarticular spongiosa.

Focal cartilage defect models can be created in a variety of small and large animals, among which the mouse, rat, rabbit, dog, minipig, sheep, goat, and horse (Hunziker 1999b; Hunziker 1999a; Hunziker 2000; Hunziker 2009). Major considerations of focal articular cartilage defect models are summarized in Table 4. Here, not only the cartilage thickness is important, but also cartilage microstructure such as cellularity (Aigner et al. 2010; Poole et al. 2010), the age of animals, defect size, depth, anatomy, and subchondral bone plate thickness (Chevrier et al. 2015). For example, a full-thickness chondral defect does not extend into the subchondral bone, but merely ends at the junction of calcified cartilage and the subchondral bone plate (Figure 2) (Frisbie et al. 2006b; Hunziker 1999b; Madry et al. 2010).

**Table 4:** **Major considerations for focal articular cartilage defect models**

**Factor**	**Comments**
Cartilage thickness	Generally increasing with the size of the animals. Depends on anatomic location within the joint
Subchondral bone plate thickness	Not always reflective of the size of the animals. Minipigs, for example, have a thin subchondral bone plate, while sheep have a thick subchondral bone plate
Age of animals	Adult animals are preferred as juvenile animals have a higher degree of spontaneous repair
Defect size	Can be determined as area of defect and placed in relation with the condylar width
Defect depth	Needs to be adapted to the osteochondral anatomy to reflect the desired defect type
Defect anatomy	Circular or rectangular patterns are commonly used
Defect location	Topographic differences within a joint exist for cartilage thickness, biochemical composition and repair potential
Knee resting position	Differs among animals, often lack of full extension as in humans
Gait patterns	Differs among animals, the sheep/goat/horse usually considered to best resemble be situation in humans

Goat, sheep, minipigs, and horses are well suited to induce chondral defects (Hunziker 1999b; Hunziker 1999a; Hunziker 2000; Hunziker 2009). Particular attention has to be paid to a meticulous surgical technique when creating chondral defect models, as the defect should not extend into the subchondral bone (Frisbie et al. 2006b; Frisbie et al. 2006a; Drobnic et al. 2010). Reflecting this important point, displaying in a publication a photomicrograph of a histological section of the chondral defect resulting from the surgical technique applied in an *ex vivo* setting of the animal model might, in some cases, be helpful to see how deep the created lesion extends. The spontaneous repair of chondral defects is rather limited, as Hunziker and Rosenberg have shown that only a few cells from the synovial membrane migrate into the lesion over time (Hunziker & Rosenberg 1996). In contrast, when marrow stimulation is applied at the base of those defects by Pridie drilling (Smillie 1957; Pridie 1959), microfracture (Steadman et al. 2001), or abrasion arthroplasty (Johnson 2001), pluripotent progenitor cells from the subchondral bone marrow migrate into the defect, differentiate into chondrocytes, and form a cartilaginous repair tissue (Frisbie et al. 1999; Frisbie et al. 2003; Shapiro et al. 1993). Interestingly, articular cartilage repair depends also on the topographic location of the defect. Clinical investigations indicate that the femoral condyles and the trochlear groove are the key locations where articular cartilage defects occur (Hjelle et al. 2002; Curl et al. 1997), and that small chondral lesions in the human femoral condyles repair better than in the trochlea (Kreuz et al. 2006b). This finding is not always reflected in the animal models. For example, articular cartilage repair in sheep is better at the trochlea than at the condyle, and therefore the repair pattern of the sheep trochlea is more reflective of the human femoral condyle (Orth et al. 2013c).

A recent focus has been to simultaneously investigate the subchondral bone in the context of osteochondral repair (Chen et al. 2011; Chen et al. 2009; Hoemann et al. 2012; Madry 2010; Goebel et al. 2012; Orth et al. 2013a; Orth et al. 2013b; Orth et al. 2012b). Data from large animal models have shown that subchondral bone changes such as the upward migration of the subchondral bone plate, intralesional osteophytes, subchondral bone cysts, and a generalized impairment of the osseous microarchitecture below the defect persist for a longer period of time than previously acknowledged (Orth et al. 2013a). Osteochondral defects, in contrast to partial- or full-thickness chondral defects, heal with the sequence of osteochondral repair (Orth et al. 2012a; Shapiro et al. 1993). Current translational studies have shown a variety of subchondral bone changes which are associated with osteochondral repair, among which the upward migration of the subchondral bone plate, the formation of intralesional osteophytes, the appearance of subchondral bone cysts, and generalized changes of the subchondral bone microarchitecture (Qiu et al. 2003; Orth et al. 2013a; Chen et al. 2009; Chen et al. 2011). These spatial and temporal alterations proceed in a defined manner (Orth et al. 2012a). Clinical studies in patients have identified similar changes of the subchondral bone below a cartilage lesion (Niemeyer et al. 2015; Bert 2015; Gomoll et al. 2010). These chiefly include the upward migration of the subchondral bone plate, intralesional osteophytes, and subchondral bone cysts (Orth et al. 2013a; Niemeyer et al. 2015; Bert 2015). Whether their development over the long-term shows a similar pattern as seen in animal models remains to be elucidated. Also, the relationship of these alterations to the degradation of the cartilaginous repair tissue remains elusive. While many animal studies did not find a correlation between the structural quality of the cartilaginous repair tissue and the advancement of the subchondral bone plate, our current understanding on the (long-term) clinical effect of such subchondral bone changes is even more limited (Niemeyer et al. 2015; Bert 2015; Sansone et al. 2015; Orth et al. 2013a; Cole et al. 2011; Vasiliadis et al. 2010; Brown et al. 2004; Kreuz et al. 2006a; Mithoefer et al. 2005; Saris et al. 2009; Dhollander et al. 2011; Henderson & La Valette 2005).

OA models are mainly established in the mouse, rat, guinea pig, rabbit, sheep, goat, and horse (Little & Zaki 2012). Notably, the Osteoarthritis Research Society International (OARSI) devoted an entire issue of their journal Osteoarthritis Cartilage for recommendations for the use of animal models in the study of OA (Johnstone et al. 1998; Agung et al. 2006). In general, utmost importance has to be given to the selection of the method for an OA induction, as this should reflect the clinical entity which is studied (Cook et al. 2010; Gerwin et al. 2010; Glasson et al. 2010; Kraus et al. 2010; Laverty et al. 2010; Little et al. 2010; McIlwraith et al. 2010). Moreover, the time of therapeutic intervention has to be carefully selected following or during OA induction.

To evaluate experimental osteochondral repair, a large variety of methods can be used and should be applied simultaneously. These include, but are not limited to macroscopic evaluation of the repair tissue and the treated joint (Goebel et al. 2012), non destructive structure evaluation by high field MRI (Goebel et al. 2014) and micro CT (Eldracher et al. 2014), biochemical (Kiss et al. 2014) and molecular biological evaluation of the repair tissue (Cucchiarini & Madry 2014), and histological (Orth et al. 2012c), and immunohistochemical evaluations (Madry et al. 2013a). Histological scoring remains the gold standard (Getgood et al. 2014). Here, both elementary and comprehensive histological scores are well suited to quantify the structure of cartilaginous repair tissue (Orth et al. 2012c). Interestingly, when evaluating osteochondral repair, only the Sellers and the Pineda score allow for an assessment of the osteochondral junction in subchondral bone (Sellers et al. 1997; Pineda et al. 1992). Close attention should be paid to sample size requirements, as a bilateral research design (where cartilage defects are established in both joints of the same animal) reduces sample sizes (Orth et al. 2013d). Recently, high field MRI at 9.4 Tesla has been shown to correlate with macroscopic and histological scoring (Goebel et al. 2014; Goebel et al. 2012). When performing micro CT analysis of the subchondral bone, a separation of the subchondral bone into subchondral bone plate and subarticular spongiosa, based on their anatomy, appears useful (Orth et al. 2012b). In the context of OA, similar principles apply, with the additional focus of the evaluation of the synovial membrane (Pastoureau et al. 2010).

### Clinical application of a magnetic device for targeted cell delivery in cartilage repair

The clinical application of a magnetic device for targeted cell delivery in cartilage repair serves as a suitable example of how data from animal models are directly translated into clinical cartilage repair (Cucchiarini et al. 2014). Current cartilage repair techniques do suffer from two weak points (Johnstone et al. 2013). One weak point is that the number of mesenchymal stem cells (MSCs) obtained in the knee with an arthroscopic procedure is limited (Min et al. 2013). The simplest strategy to increase the number of the cells is the intra-articular injection of MSCs after increase of autologous MSCs by cultivation (Frisch et al. 2014). MSCs are the cell population of undifferentiated cells isolated from adult tissue that have the capacity to differentiate into mesodermal lineages, such as bone, cartilage, fat, muscle or other tissues (Johnstone et al. 1998; Johnstone & Yoo 1999). MSCs from the bone marrow can be cultured and differentiated into the desired lineage *in vitro* with the application of specific growth factors or bioactive molecules (Zellner et al. 2014). Intra-articular injection of too many MSCs, however, generated free bodies of scar tissue (Agung et al. 2006). A novel stem cell delivery system for cartilage repair using magnetically labelled MSCs and an external magnetic device was therefore developed, aiming at accumulating a relatively small number of MSCs to a desired location. Ferumoxides are dextran-coated superparamagnetic iron oxide nanoparticles approved by the US Food and Drug Administration (FDA) as a magnetic resonance contrast agent by intravenous injection for hepatic imaging of humans (Wang et al. 2001). By employing ferumoxides, it is easy to generate magnetically labelled MSCs. The ability to deliver magnetically labelled MSCs to a cartilage defect that is a desired place under arthroscopy was demonstrated in rabbit and swine knee joints using an external magnetic device at 0.6 Tesla (Kobayashi et al. 2008).

This result indicates that this minimally invasive system under arthroscopy can be applicable for a focal osteochondral defect in the knee joint. The next step was to examine if this external magnetic system is effective for OA. Here, the question was investigated if a cartilage layer on degenerated human cartilage could successfully be regenerated *in vitro* using this external magnetic system (Kobayashi et al. 2009). MSCs from human bone marrow were cultured and magnetically labelled. Degenerated human cartilage was obtained during total knee arthroplasty. The osteochondral fragments were attached to the sidewall of tissue culture flasks, and magnetically labelled MSCs were injected into the flasks. Using an external magnetic device, a magnetic force was applied for 6 h to the direction of the cartilage, and then the degenerated osteochondral fragment was cultured in chondrogenic differentiation medium for 3 weeks. In the control group, a magnetic force was not applied. Histological evaluated revealed that a new cell layer has formed on the surface of the degenerated cartilage. Positive staining of this cell layer with Toluidine blue, Safranin O, and with anti-type-II collagen immunostaining indicated that is contained a cartilage-like extracellular matrix. In the control group, such a cell layer was not observed. In conclusion, these findings demonstrate that the magnetic system can deliver MSCs onto degenerated human cartilage, which then form an abundant extracellular matrix *in vitro*. Another experimental study was conducted using minipigs to further demonstrate the effectiveness of this system for cartilage repair (Kamei et al. 2013). The safety of the cell application and the capability of the cell proliferation were also investigated and confirmed (Kamei et al. 2013). The first-in-men to apply this procedure to human with a cartilage defect will be started in 2015.

Another weak point is the overload of the focal area treated by marrow-stimulation as a result of early postoperative weight-bearing (Orth et al. 2012b). A novel approach to solve this problem is to reduce the load to the repaired area to protect immature tissue regenerated at the repaired area against destruction caused by overloading. A new distraction arthroplasty device was introduced (Meira, Nagoya, Japan), which allows the range of motion with knee joint distraction (Deie et al. 2007). After an experimental study using rabbits, this technique was applied to patients with OA. After drilling or microfracture under arthroscopy, the new external device was fixed with four 6-mm pins drilled into the distal femur and the proximal tibia. After the appropriate distractive tension was applied, the ROM and the post-distraction and pre-distraction tibiofemoral joint spaces at 30° of flexion were measured. Although this device is usually applied for 3 months, full weight bearing is allowed one month after surgery. Until now, this device has been demonstrated to function well to repair human articular cartilage defects.

## Conclusions

The complexity of reconstructive surgical approaches for musculoskeletal disorders complicates the development of novel therapies. Large animal models continue to play a major role to translate new surgical techniques to patients. Although there is no single large animal model replicating the human knee joint perfectly, the sheep stifle joint shares strong similarities with the human knee joint. Large animal models for the treatment of ligamentous ACL lesions may help to overcome the therapeutic dilemma of how to treat such tears in the paediatric population. The newly developed preclinical large animal model of HTO can be applied to evaluate different types of osteotomies and to the effect of lower limb alignment on the reconstructive cartilage therapy. Animal models for cartilage repair need to precisely reflect the clinically very different aetiologies of articular cartilage defects, appearances and appropriate surgical options. This results in the currently large variety of different animal models for focal defects and OA. In the clinical situation, the combination of injection of magnetically labelled MSCs under magnetic field and external arthroplasty device may be an option to successfully treat large osteochondral defects or OA in the near future. As novel insights from studies using these translational models will advance the basic science, and close cooperation in this important field of clinical translation (Madry et al. 2014a) will improve current reconstructive surgical options, the clinical application of such innovative approaches will open novel avenues for regenerative therapies of musculoskeletal disorders.
